# Thymidine phosphorylase facilitates retinoic acid inducible gene-I induced endothelial dysfunction

**DOI:** 10.1038/s41419-023-05821-0

**Published:** 2023-04-26

**Authors:** Adrian Baris, Eugenia Fraile-Bethencourt, Jaiden Eubanks, Sokchea Khou, Sudarshan Anand

**Affiliations:** 1 Department of Cell, Developmental & Cancer Biology, Knight Cancer Institute, 2720 S Moody Avenue, Oregon Health & Science University, Portland, OR 97201 USA; 2 Department of Radiation Medicine, Knight Cancer Institute, 2720 S Moody Avenue, Oregon Health & Science University, Portland, OR 97201 USA

**Keywords:** Apoptosis, Stress signalling

## Abstract

Activation of nucleic acid sensors in endothelial cells (ECs) has been shown to drive inflammation across pathologies including cancer, atherosclerosis and obesity. We previously showed that enhancing cytosolic DNA sensing by inhibiting three prime exonuclease 1 (TREX1) in ECs led to EC dysfunction and impaired angiogenesis. Here we show that activation of a cytosolic RNA sensor, Retinoic acid Induced Gene 1 (RIG-I) diminishes EC survival, angiogenesis and triggers tissue specific gene expression programs. We discovered a RIG-I dependent 7 gene signature that affects angiogenesis, inflammation and coagulation. Among these, we identified the thymidine phosphorylase TYMP as a key mediator of RIG-I induced EC dysfunction via its regulation of a subset of interferon stimulated genes. Our RIG-I induced gene signature was also conserved in the context of human diseases – in lung cancer vasculature and herpesvirus infection of lung endothelial cells. Pharmacological or genetic inhibition of TYMP rescues RIG-I induced EC death, migration arrest and restores sprouting angiogenesis. Interestingly, using RNAseq we identified a gene expression program that was RIG-I induced but TYMP dependent. Analysis of this dataset indicated that IRF1 and IRF8 dependent transcription is diminished in RIG-I activated cells when TYMP is inhibited. Functional RNAi screen of our TYMP dependent EC genes, we found that a group of 5 genes - Flot1, Ccl5, Vars2, Samd9l and Ube2l6 are critical for endothelial cell death mediated by RIG-I activation. Our observations identify mechanisms by which RIG-I drives EC dysfunction and define pathways that can be pharmacologically targeted to ameliorate RIG-I induced vascular inflammation.

## Introduction

The vascular endothelium is an incredibly complex, dynamic system with a variety of functions in cardiovascular health. As such, endothelial cells (ECs) can face numerous injuries that lead to pathogenesis, disease, and dysfunction. Understanding how ECs undergo dysfunction and methods to target that dysfunction are critical to understanding and improving cardiovascular disease treatment. There is emerging evidence that nucleic acid sensors (NAS)—one of the fundamental arms of innate immunity—play a critical role in endothelial cell function [1–10].

Among NAS, RIG-I recognizes RNA cytosolic 5’ppp short RNAs. Upon recognition of RNA, the base-paired region of RNA complexes with the HD of RIG-I, released the CARD, which is normally bound to the HD in a repressing form. This interaction displaces CARDs, which causes several RIG-I proteins to oligomerize and become accessible for MAVS signaling. RIG-I binds to MAVS through homotypic CARD-CARD interactions. Once activated, MAVS recruits the tumor necrosis factor receptor associated factors, which activate interferon regulatory factors 3 and 7, and the NF-kB mediated pathway. This results in the expression of cytokines and IFN-I genes, which recruit innate and adaptive immune cells [11–13].

It has been shown that RIG plays a role in EC dysfunction and inflammation [6]. In addition, it is well known that ECs are the site of cytokine storms following infection, and that inflammation in ECs from innate immune sensing can impact tumor growth [14]. Here we show that RIG-I activation using a small 5’ppp RNA diminishes EC survival and causes EC dysfunction. We have discovered a RIG-I dependent 7 gene signature that affects angiogenesis, inflammation and coagulation. Among these, we identified the thymidine phosphorylase TYMP as a key mediator of RIG-I induced EC dysfunction via its regulation of a subset of interferon stimulated genes. Inhibition of TYMP with a small molecule drug or siRNA rescues RIG-I induced EC death, migration arrest and restores sprouting angiogenesis likely via decrease of IRF1 dependent transcription. Our observations identify mechanisms by which RIG-I drives EC dysfunction and define pathways that can be pharmacologically targeted to ameliorate RIG-I induced vascular inflammation. Our studies identify novel mechanisms by which RIG drives endothelial dysfunction and highlights potential strategies for mitigating inflammation caused by RNA sensing.

## Results

### Endothelial RIG-I activation causes EC dysfunction

**Fig. 1:**
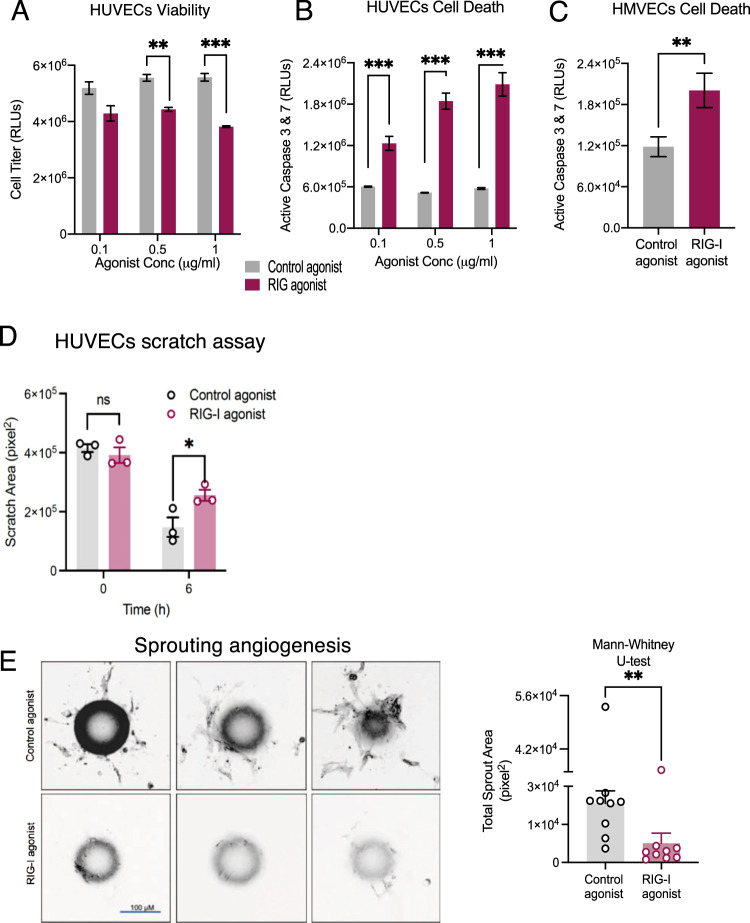
RIG-I activation causes EC dysfunction. HUVECs or HMVECS were treated with either control agonists or RIG-I agonists (1 ug/ml) (A-E). Viability was measured using Cell Titer glo (**A**) and Cell Death was measured using Caspase 3&7 glo (**B**–**C**) assays. **D** Migration was measured using a scratch assay. **E** Sprouting angiogenesis was measured using a 3D tube formation assay in a fibrin gel. Scale bar = 100 μm. Quantification of sprout area stained using *U.europaeus* lectin. Dots represent individual beads. **P < 0.01, ***P < 0.005 using ANOVA or two-tailed Student’s *T*-test and Mann–Whitney *U*-test for (**E**). Bars represent mean ± SEM of independent replicates.

It is well understood that RIG-I can be robustly activated using a small hairpin triphosphate containing RNAs. We have previously established a 89 base-paired RNA agonist from the influenza virus is a potent activator of RIG-I signaling [15]. To determine the effect of RIG-I activation on ECs, we treated HUVECs and HMVECs with a control agonist (14 base paired ds RNA) or a RIG-I agonist, validated the increase in interferon stimulated genes (Supplementary Fig. 1) and evaluated EC health. We found the RIG-I agonist significantly decreased proliferation and increased cell death in a dose responsive manner (Fig1A–1C). To further understand the functional consequences of RIG-I agonist treatment, we used a scratch assay. We identified significant delays in wound healing upon treatment with a RIG-I agonist (Fig. 1D). Similarly, we found that upon transfection of the RIG-I agonist, endothelial cells were unable to form angiogenic sprouts in a 3D fibrin bead assay (Fig. 1E). These observations suggest that activation of RIG-I impacts EC proliferation, survival, and fundamental functional characteristics such as migration and sprouting angiogenesis.

### RIG-I drives a specific angiogenic gene signature

**Fig. 2:**
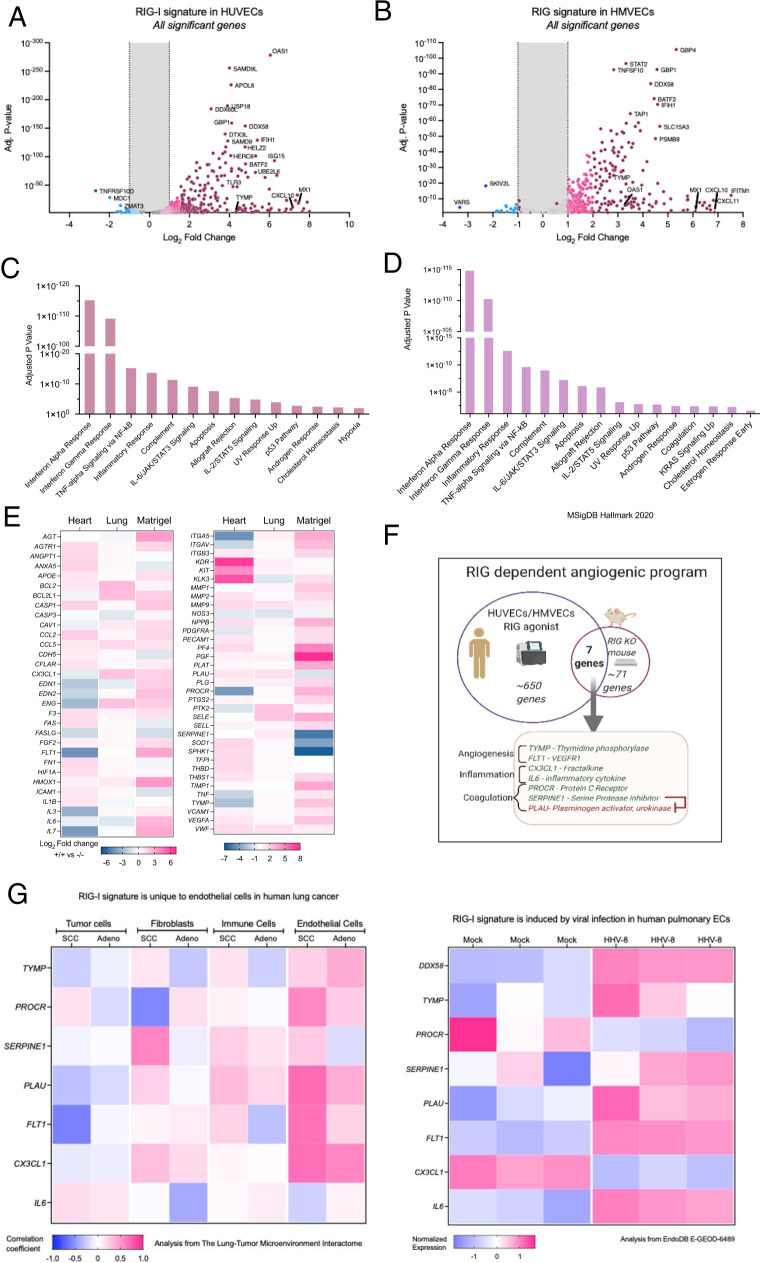
RIG-I drives a specific angiogenic gene signature in ECs. **A**–**C** HUVECs or **B**–**D** HMVECs were treated with RIG agonists or control agonist. 24 h later RNA was extracted and RNA sequencing was performed. Data was analyzed using RaNAseq pipeline as described in Prieto & Barrios, *Bioinformatics* 2020. All genes with adjusted *P*-values <0.05 are plotted and critical IFN responsive genes are colored and labeled. **C**–**D** Gene set enrichment analysis using Enrichr. **E** Heatmap depicting gene expression changes in indicated tissues from WT vs RIG-I −/− mice (*n* = 2) from an angiogenesis qRT-PCR array. **F** Schematic depicting a 7 gene RIG-I dependent angiogenic signature. **G** Heatmap depicting enrichment of endothelial RIG-I signature from F in public datasets. Left panel depicts correlation coefficients of RIG-I with the 7 genes across four major cell types from the lung-tumor microenvironment interactome, a dataset of scRNA-seq from patients with squamous cell carcinoma or adenocarcinoma of the lung. Right panel depicts normalized expression scores of RIG-I and the 7 gene signature in human pulmonary ECs during infection with human herpesvirus 8 (HHV-8) plotted from EndoDB dataset E-GEOD-6489.

To identify transcriptional changes involved in RIG-I activation, preformed RNA sequencing from control or RIG-I activated HUVECs and HMVECs 24 h post treatment. We found that RIG-I activation with this agonist led to a robust interferon response, including an upregulation of genes such as MX1, OAS1, and CXCL10. In addition, we also found that DDX58, the gene that encodes RIG-I, is upregulated following RIG-I activation, indicating a feed-forward loop (Figs. 2A, 2B). We performed a pathway enrichment analysis using Enrichr [16, 17] and found that type I interferon signaling is highly enriched (Figs. 2C, 2D). While pathway analysis inferred interferon gamma response, endothelial cells do not make interferon gamma. The Enrichr annotation for this pathway consists of 39 genes including Akt1, IL-1B, IRF1, JAK1, STAT1, STAT3, several MAP kinases that are common to other pathways induced by RIG-I activation. In addition to these canonical pathways, we observed pathways that are important for vascular function such as cholesterol homeostasis, coagulation and hypoxia were modulated by RIG-I activation (Figs. 2C, 2D).

To complement our gain of function studies in human cell lines, we performed a targeted angiogenesis gene signature evaluation from RIG-I deficient mice. We obtained a RIG-I gene edited mouse (Supplemental Fig. 2). While these mice are viable and fertile, we observed that they had more vascular leak in a bFGF Matrigel plug model (Supplemental Fig. 2). To identify transcriptional changes in these mice, we harvested the Matrigel, heart and lungs from RIG-I (+/+) and (−/−) mice. Using a qPCR angiogenesis array, we found several organ-specific angiogenic changes in RIG-I (−/−) mice (Fig. 2E), which is indicative of RIG-I’s role in angiogenesis. Finally, we compared our human gain of function and mouse loss of function datasets and discovered a unique gene signature that consists of 7 genes that are commonly regulated in both human and mouse (Fig. 2F). We validated our 7 gene signature using independent qPCR assays (Supplemental Fig. 3). These 7 genes are known to play a role in angiogenesis and coagulation. We observed that our gene signature is also highly correlated with RIG-I expression only in the endothelial cells in squamous cell carcinomas and adenocarcinomas of the lung based on a dataset of single cell RNAseq from human lung cancer patients[18] (The Lung-Tumor Microenvironment Interactome) (Fig. 2G). Similarly, analysis of a public dataset of human Herpesvirus 8 infected pulmonary endothelial cells from EndoDB [19] also revealed high normalized expression of RIG-I as well as the 7 signature genes. We observed some differences between our data and the public datasets. We observed PLAU was downregulated with RIG-I activation whereas it was upregulated in the lung adenocarcinoma endothelial cells. Similarly, PROCR and CX3CL1 also decreased in the Herpesvirus treated ECs whereas we observed these genes are induced with RIG-I activation. Therefore, there maybe context specific regulation of genes in our RIG-I signature.

Thymidine phosphorylase (TYMP) and VEGFR1 are known regulators of angiogenesis [20, 21]. Fractalkine and interleukin-6 and heavily involved in inflammation [22, 23]. A recent proteomics study showed that patients with fatal COVID19 have enhanced expression of both TYMP and RIG-I in lung parenchyma with TYMP being the most significantly induced gene [24]. In addition, Protein C Receptor and Serine Protease inhibitor are regulators of coagulation[25, 26]. Interestingly, Plasminogen activator urokinase is negatively regulated by SERPINE1, and is downregulated by RIG-I activation. We chose to focus on TYMP due to its high expression in human ECs following RIG-I activation.

### TYMP facilitates RIG-I induced EC dysfunction

**Fig. 3:**
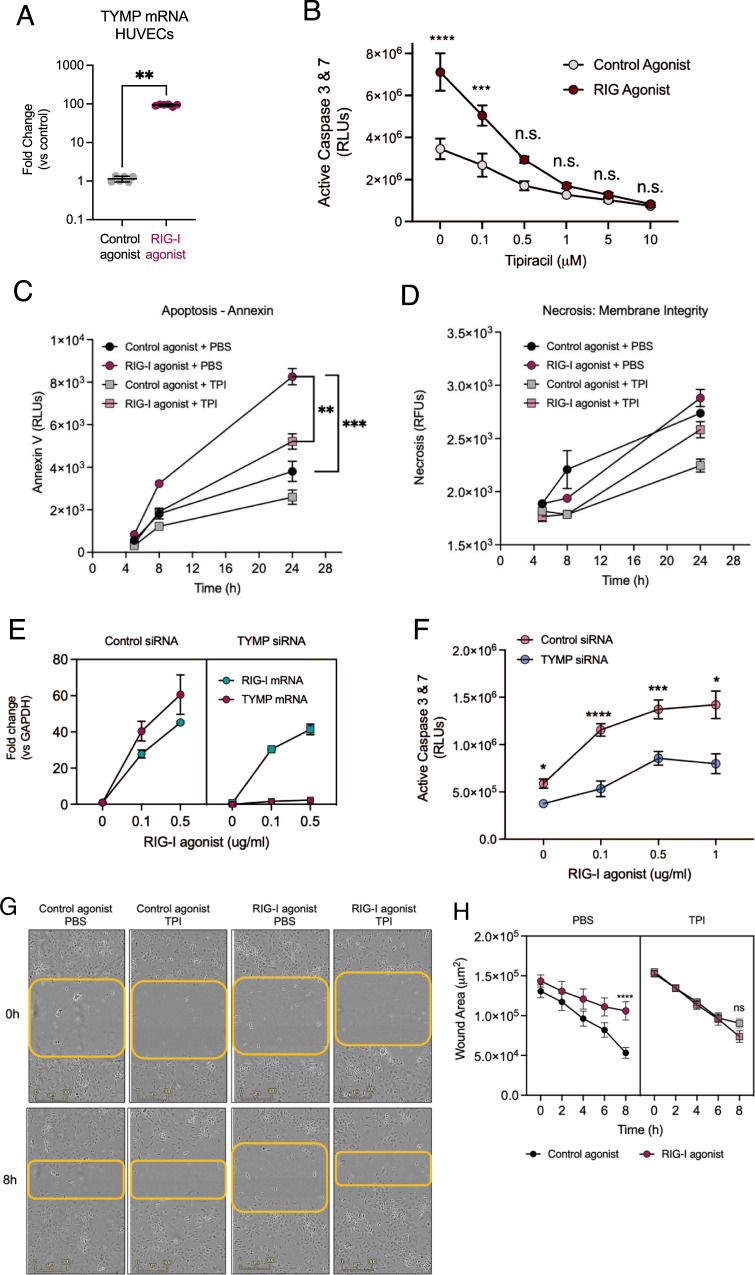
TYMP inhibition partially rescues RIG-I activation induced phenotypes. **A** TYMP induction was measured using qRT-PCR. **B** HUVECs were treated with a control or RIG-I agonist in combination with Tipiracil. **B** Cell Death was assessed using a Caspase Glo assay (**C**) Annexin V luminescence assay and (**D**) Necrosis assay. **E**–**F** HUVECs were transfected with either a control siRNA or TYMP siRNA and treated with a control agonist or RIG-I agonist at the indicated doses. **E** mRNA levels showing efficient knockdown of TYMP 24 h post transfection. **F** Cell death was measured using Caspase-Glo assay. **G**–**H** Migration was assessed using a scratch assay in a 6-well TC plate. Scale bar = 100 uM. *P < 0.05, **P < 0.01, ***P < 0.005, ****P < 0.001 using ANOVA or two-tailed Student’s *T*-test. Bars represent mean ± SEM of independent replicates. One of two independent experiments.

We identified TYMP as being the most upregulated gene by RIG-I activation in HUVECs. Notably, TYMP is strongly upregulated at the mRNA and protein levels in RIG-I activated HUVECs (Fig. 3A and Supplementary Fig. 3B). In addition, high levels of TYMP in tumor endothelium are linked to increased angiogenesis and a poor prognosis [27–30]. The TYMP inhibitor Tipiracil (TPI), is commonly used in anti-cancer treatments to prevent the degradation of 5-FU and has been shown to prevent thrombosis [31].

To evaluate the role of TYMP in RIG-I induced EC dysfunction, we tested the impact of TPI treatment on RIG-I induced phenotypes. We found that TPI prevents RIG-I associated apoptosis in a dose-dependent manner (Fig. 3B). We observed increase in Annexin V staining as early as 8 h post RIG-I activation. This increase in Annexin staining was also diminished by TPI treatment (Fig. 3C). In contrast to the caspase and Annexin readouts of apoptosis, we did not observe any appreciable effect of RIG-I on necrosis (Fig. 3D). To complement the effects of pharmacological TYMP inhibition, we used siRNA to knockdown TYMP. We first confirmed that siRNA was able to diminish TYMP levels even during the robust induction with the RIG-I agonist (Fig. 3E and Supplementary Fig. 3C). Similar to our TPI studies, TYMP siRNA decreased the induction of caspases 3 & 7 (Fig. 3F). In addition, we found that TPI reverses RIG-I induced migration arrest (Fig. 3G–H). Complementary to the pharmacological inhibition, siTYMP also rescued RIG-I induced migration arrest (Supplementary Fig. 4). This indicates that TYMP may have specific roles in disrupting EC viability, and migration downstream of RIG-I activation. Therefore, pharmacological inhibition of TYMP might mitigate endothelial dysfunction.

### Identification of putative TYMP dependent genes and pathways that regulate RIG-I function

**Fig. 4:**
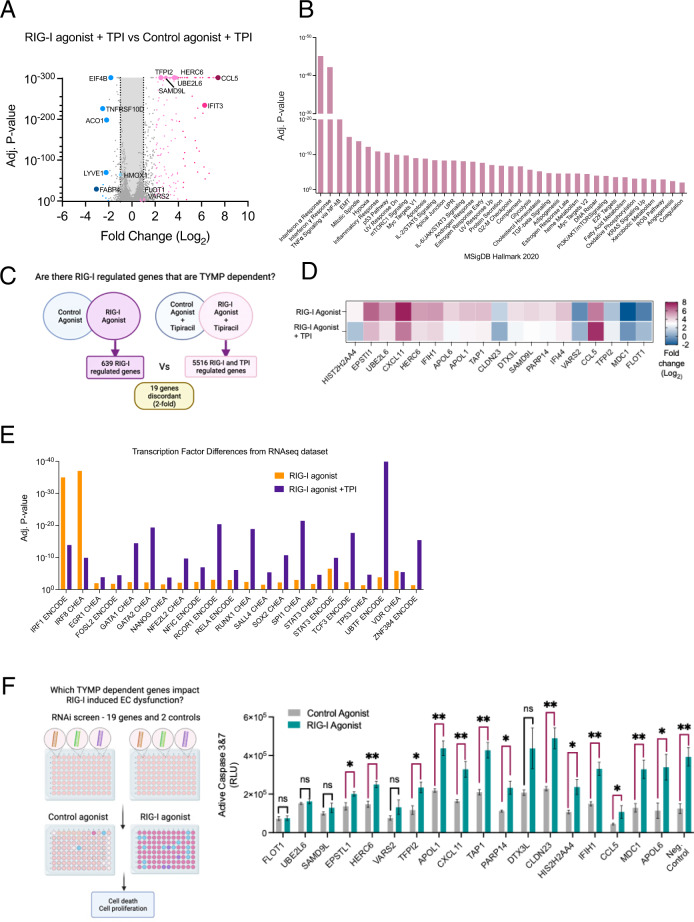
TYMP dependent genes that impact RIG-I induced EC dysfunction. **A**, **B** HUVECs were treated with control agonist or RIG-I agonist in combination with Tipiracil or vehicle (PBS). RNAseq was performed 24 h after treatment. **A** Volcano plot depicting differentially expressed genes (**B**) Gene set enrichment analysis (Enrichr) is depicted. **C**, **D** 19 genes were identified as differentially expressed (ie up with RIG-I but down with RIG-I + TPI or vice-versa). **E** Consensus transcription factor analysis from ENCODE and ChEA (Enrichr) (**F**) HUVECs were transfected with 4 pooled siRNAs against each of the 19 genes. 24 h later cells were treated with control or RIG-I agonist and cell death was measured. Loss of significance in bar graph indicate the siRNAs that prevent RIG-I induced EC death. One of two independent experiments.

To identify the mechanism behind TPIs reversal of RIG-I activation phenotype in HUVECs, we performed RNA-seq on control and RIG-I activated HUVECs with and without TPI treatment (Fig. 4A–B). We identified 19 genes that were differentially regulated (at least 2-fold) in TPI RIG-I activated HUVECs compared to RIG-I activation alone (Fig. 4C–D). We further evaluated the transcription factor motif enrichment in our RNAseq data and found that IRF1 and IRF8 motifs were diminished in the RIG-I agonist + TPI treated cells compared to the RIG-I agonist treatment alone (Fig. 4E). We evaluated whether the gene signatures were representative of a direct effect on IRF levels. We observed that RIG-I treatment led to increased expression of IRF1 and not IRF8 in HUVECs (Supplementary Fig. 5A). The induction of IRF1 was lost upon TPI treatment. We assayed for the expression of three known IRF1 target genes – CXCL9, CXCL10 and CXCL11. While TPI decreased RIG-I induced CXCL9 and CXCL11, there was a robust increase in the levels of CXCL10 upon RIG-I activation combined with TPI treatment (Supplementary Fig. 5B). These observations suggest that although IRF1 is induced by RIG-I and suppressed by TPI, there are complex interactions either in terms of nuclear localization, engagement with other transcription factors/co-factors etc. that dictate which subset of IRF1 transcribed genes are RIG-I/TYMP dependent.

To evaluate the functional significance of our 19-gene-signature, we then performed a limited siRNA screen in control or RIG-I activated conditions. We sought to test which siRNAs diminished the ability of RIG-I to drive apoptosis in HUVECs. (Fig. 4F). We found siRNAs targeting 4 TPI regulated genes – FLOT1, UBE2L6, SAMD9L, VARS2 diminished RIG-I induced cell death. It is possible that the interference of ISG signaling, due to the downregulation of these genes via decreased IRF1 or IRF8 is responsible for TPI’s ability to reverse the RIG-I phenotype. Our observations of putative mechanism(s) by which TYMP regulates endothelial function in the context of RIG-I activation need further work to provide definitive mechanistic hypotheses.

In summary our results demonstrate that RIG-I is a potent driver of EC death, diminishes migration and sprouting angiogenesis. We also identify RIG-I induced genes in vitro and RIG-I dependent genes in vivo and show that a thymidine phosphorylase TYMP mediates some of the biological effects of RIG-I.

## Discussion

Studies have shown that chronic activation of the immune system, such as from pattern recognition receptors (PRRs) can lead to adverse changes in vascular structure and function[32]. There is evidence that activation of the viral RNA sensor RIG-I induces endothelial dysfunction, vascular oxidative stress, and the recruitment of innate immune cells. RIG-I activation has been observed in several inflammatory and autoimmune disorders. A clear example of this is in Singleton-Merten Syndrome, which is caused by a mutation leading to constitutively active RIG-I [33, 34]. Singleton Merton Syndrome leads to several vascular defects such as aortic calcification and skeletal abnormalities. While there are pathological consequences associated with RIG-I signaling, activation of RIG-I is also being explored as a promising therapeutic strategy in cancer. Therefore, understanding how RIG-I causes EC dysfunction and approaches to mitigate the vascular pathologies associated with RIG-I activation become important.

Our work determined that RIG-I activation can induce angiogenic defects in endothelial cells. Specifically, we observed both HUVECS and HMVECs had significant apoptosis but not necrosis in response to our RIG-I agonist and decreased migration, sprouting angiogenesis. Asdonk et al [6] have also found that RIG-I activation leads to an increase in vascular oxidative stress and reactive oxygen species in vivo and in vitro. While Asdonk et al identified vascular dysfunction in human coronary endothelial cells (HCAEC) and endothelial progenitor cells (EPC), our studies highlight that this affect occurs across a wide range of endothelial cell types and that RIG-I activation induces a wide range of phenotypes associated with EC dysfunction [6]. While our in vivo studies in the knockout mice (Supplementary Fig 2) demonstrate an increase in hemoglobin content, this could potentially be due to increased vascular leak or increased angiogenesis or a decrease in coagulation/thrombosis. These observations establish RIG-I activation as a robust driver of EC dysfunction.

To understand the biological and mechanistic basis for RIG-I activation induced phenotypes, we performed RNAseq experiments from our cell culture models and an angiogenesis focused qRT-PCR array from Matrigel plugs from our RIG-I^−/−^ mice. Using gene set enrichment analysis tools, we found significant enrichment of interferon signaling pathways, as well as cholesterol homeostasis, coagulation and hypoxia pathways in both HUVECs and HMVECs. Many of these pathways are directly relevant to the phenotypes we observed in Fig. 1. Comparing the human EC gain of function and the mouse tissue EC’s loss of function signatures, we deduced a unique 7 gene signature of RIG-I activation. Our signature appears to be relevant in human disease pathologies. For example, we found that our 7 genes showed high positive correlations with RIG-I expression in the endothelial cells from lung cancer, both squamous cell carcinomas and adenocarcinomas [18]. Similarly, we analyzed a publicly available dataset and observed that RIG-I as well as the 7 gene signature was upregulated in lung endothelial cells in response to HHV-8 infection. TYMP and RIG-I were recently identified as part of a 22-protein signature associated with fatal COVID-19 in a proteomics study [24].

Thymidine phosphorylase (TYMP) is abundantly expressed in platelets and induces EC migration, inhibits apoptosis, and has been shown to stimulate pro-angiogenic factors such as VEGF in a context-dependent manner. Till date there is some evidence indicating TYMP plays a pro-angiogenic role in endothelial cells and endothelial progenitor cells [35]. It has been shown that loss of TYMP leads to mitochondrial neurogastrointestinal encephalopathy [36] and TYMP interacts with VEGF to promote the breakdown of the blood-brain barrier [37]. TYMP inhibition using the pharmacological inhibitor tipiracil (TPI) is used in a clinical setting and has been shown to reverse thrombosis [31]. We found that TPI is able to rescue HUVECs from RIG-I induced cell death and restore the migration abilities of RIG-I activated HUVECs. An siRNA recapitulated the phenotype by protecting HUVECs from RIG-I induced cell death. These observations indicate that TYMP is a positive regulator of RIG-I induced EC dysfunction. Indeed, proteomics studies have identified TYMP and RIG-I as being highly upregulated in lungs of patients with lethal COVID infections[24]. Our RNAseq data from RIG-I activated ECs with and without TPI identified a robust group of 19 genes that were RIG-I induced and TYMP dependent. Our subsequent RNAi experiment highlighted five of these genes as being functionally relevant for RIG-I induced cell death. Two of these genes, UBE2L6 and SAMD9L, are associated with the ISG signaling pathway [38, 39]. VARS2 is a mitochondrial gene whose mutation is linked to mitochondrial encephalopathies. The gene FLOT1 is implicated in proliferation and tumorigenicity [40]. CCL5 is a chemokine involved in inflammatory responses [41]. As we observed in our gene set enrichment analysis, IRF1 and IRF8 transcription factor motifs appear to be diminished during TPI treatment. While we do observe IRF1 induction with RIG-I and loss with TPU treatment, it remains to be seen if these transcription factors are directly responsible for the induction of RIG-I induced, TYMP dependent ISGs that lead to endothelial cell death. One limitation of our data is that these observations of TYMP dependent gene expression pathways are correlative. Additional functional studies are necessary to elucidate the mechanisms by which TYMP and RIG-I regulate EC dysfunction.

Our work here indicates that pharmacological inhibition of TYMP using TPI reverses a subset of RIG-I induced genes and phenotypes in cultured ECs, creating a protective effect. We have also identified a subset of ISG-related genes that are inhibited by TPI in a RIG-I activated context, identifying a potential mechanism for TYMP’s reversal of the RIG-I activated angiogenic defects. Taken together, our findings highlight novel pathways that drive RIG-I induced EC dysfunction and provide potential druggable targets that can mitigate some of the cardiovascular pathologies associated with RIG-I activation.

## Materials and methods

### Cell culture and reagents

Human umbilical vein endothelial cells (HUVECs) (Cat: C-2519, Lonza) and dermal human microvascular endothelial cells (HMVECs) (Cat: CC-2543, Lonza) were cultured in EBM-2 media (Cat: NC1447083, Fischer) supplemented with bullet kit and 10% fetal bovine serum (Cat: S11550H, Biotechne). Normal human lung fibroblasts (Cat: CC-2512, Lonza) were cultured in FBM media (Cat: CC3131, Lonza) and supplemented with bullet kit and 10% fetal bovine serum. All cells were maintained at 37 °C and 5% CO_2._ Cells used for experiments were low passage number between 2–8. Tipiracil (TPI) was purchased from Sigma (Cat: SML1552-10MG) and dissolved in PBS. Cell lines were authenticated with STR profiling if maintained in the lab (not purchased). All cell lines were routinely tested and found negative for mycoplasma contamination before use in the assays as described [42].

### Cell transfection

HUVECs (70% confluence) were transfected with 0.1–1 ug/mL RIG-I or control agonist RNA (Cat: tlrl-hprna-100, Invivogen) according to manufacturers’ instructions. Specifically, The RIG-I or control agonist was diluted in Lyovec transfection reagent (Cat: lyec-12 Invivogen).

### Cell viability and apoptosis assays

Viability and apoptosis were assessed using the Cell-Titer Glo and Caspase-Glo kits, respectively (Cat: G9242, G8091, Promega). White walled, 96-well tissue culture microplates (Corning) were used for luciferase-based assays. Luminescence was measured using a Promega GloMax instrument using a 0.5 s integration time.

### RNA sequencing and gene expression

Total mRNA was isolated from cells and tissues using the Eurx RNA isolation kit (Cat: E3598-02, EURX). RNA sequencing was performed using the Oregon Health and Sciences University Massively Parallel Shared Sequencing Resource (MPSSR) and analyzed using the RANAseq pipeline and web interface with differential expression comparisons through DESeq2 and Wald’s test for significance calculations [43]. Reverse transcription was preformed using High-Capacity cDNA Reverse Transcription Kit (Cat: 4368814, Applied Biosystems) according to manufacturer’s instructions. Gene expression was measured using real-time quantitative PCR (qRT-PCR) with TaqMan Master Mix II no UNG (Cat: 4440048, Thermofisher Scientific) with the following primers: human TYMP (Cat: 4453320, Assay ID: Hs00157317_m1), human FLT1 (Cat: 4453320) Assay ID: Hs01052961_m1, human CX3CL1 (Cat: 4448892, Assay ID: Hs01011407_m1), human IL6 (Cat: 4448892, Assay ID: Hs03929033_u1), human PROCR (Cat: 4448892, Assay ID: Hs00941183_g1), human SERPINE1 (Cat: 4453320, Assay ID: Hs00167155_m1), human PLAU (Cat: 4448892, Assay ID: Hs01547050_m1) and human GAPDH (Cat: 4331182, Assay ID: Hs02758991_g1) according to manufacturer’s instructions.

Silencer Select Pre designed siRNA siRNA ID: s4434 or Silencer™ Select Negative Control No. 1 siRNA were purchased from Qiagen. Endothelial cell biology angiogenesis gene array was assessed according to manufacturer’s instructions (Cat: 330231 PAMM-015ZE-4, Qiagen). Specifically, the SYBR Green qRT-PCR assays were conducted using PowerUP SYBR Green Master Mix with predetermined primers in the array. Fold change was calculated using the 2^−ΔΔCt^ method relative to an internal control (GAPDH).

### Western and simple western blots

HUVECs were seeded in six well plates (2,000,000 cells/well) and transfected as described above. In some cases, after 24 h, cells were treated with 10 nmol TPI for 24 h. After treatment, media was aspirated and cells were washed in ice cold PBS and lysed directly in the plate in RIPA buffer (Cat: PI89900, Fischer) containing Protease Inhibitor Mini Tablets (1/10 mL RIPA buffer, Cat: 50-720-4060, Fisher) with phosphatase inhibitor cocktail 2 and 3 (1:1000, P5726-1ML, P0044-1ML, Sigma). Lysates were centrifuges for 12,000 x g and 4 °C for 20 min. The supernatant was collected, and protein concentration was determined using the Pierce BCA Protein assay kit (Cat: 23227, Thermofisher).

For western blot, samples were mixed with 4x Protein Sample Loading Buffer (Cat: 928-40004, Li-Cor) supplemented with 5% of beta-mercaptoethanol, denaturalized and loaded in 4–20% precast polyacrylamide gels (Cat: 456-1094, BioRad). Electrophoresis was done in 1x Tris/Glycine/SDS at 200 V for 30 min. Trans-blot Turbo Transfer system (Biorad) was used to transfer the proteins to a PVDF membrane. Blocking and antibody dilutions were done in Intercept blocking buffer (Cat: P/N 927-60001, Licor). The membranes were developed using Li-Cor Odyssey Clx imaging system. For simple western blot, the samples were diluted with 1x sample buffer (Cat: DM-001, ProteinSimple). Protein quantification was performed using a 12-230 kDA 25 lane plate (Cat: SM-W004, ProteinSimple) in a ProteinSimple Wes Capillary Western Blot analyzer according to the manufacturer’s instructions. Anti-RIG-I (#3743), Anti- ISG15 (#2743), Anti-TYMP (#4307), Anti-IRF8 (#98344) and Anti- GAPDH (#5174) were all purchased from Cell Signaling. Anti-IRF1 (PA5-64093) was purchased from ThermoFisher.

### Scratch assay

HUVECs were plated on a 12 well plate at 100% confluency and the monolayer was scratched with a pipette tip. At 0, 4, and 8 h, the scratch area was visualized using brightfield microscopy in an Incucyte Live Cell Analysis Platform. Area of scratch was quantified using Image J typically by an observer blinded to the experimental groups.

### 3D angiogenic sprouting assay

HUVECs transfected for 24 h and were coated on cytodex-3 (x) beads at a density of 1 million cells per 25 uL beads and incubated in suspension for 3–4 h with gentle mixing every hour. In some cases, TPI was added during the incubation. Beads were then plated on TC treated 24 well dishes overnight and resuspended in 2 mg/ml fibrin gel with 200,000 fibroblasts. The gel was allowed to polymerize and complete EGM-2 media was added. Sprouts were visualized on day 4 via confocal imaging following a 4-h incubated with 1:200 flourescein isothiocyanate (FITC)-labelled *Ulex europaeus* lectin (Vector labs) [44].

#### In vivo methods

All animal work was approved by the OHSU Institutional Animal Use and Care Committee. WT male and female C57Bl/6 N and Ddx58 (−/−) 8–10-week-old mice were purchased from Jackson Labs and injected subcutaneously with Growth factor reduced (Matrigel BD) with 400 ng mL^−1^ recombinant human bFGF (Millipore). One-week later Matrigel plugs, as well as lung, liver, and heart, were harvested from mice and RNA was isolated using the Eurx RNA purification kit according to manufacturer’s instructions. Matrigel plugs were homogenized and analyzed for hemoglobin content using a colorimetric assay kit (Sigma). In addition, RNA from tissue was used to analyze endothelial activity using the Qiagen endothelial cell activity qRT-PCR array according to manufacturer’s instructions.

### Statistical analysis

All statistical analysis was performed using Prism software (GraphPad Software, San Diego, CA). In vitro experiments were performed in biological replicates (typically *N* = 3 unless stated otherwise in the legends). No statistical methods were used to predetermine sample size for mouse studies. No animals were excluded from analysis. No blinding or randomization was used in the studies. Differences between pairs of groups were analyzed by Student’s *t*-test. Variance was similar between groups. Comparison among multiple groups was performed by one-way ANOVA followed by a post hoc test (Tukey’s or Holm-Sidak). In the absence of multiple comparisons, Fisher’s LSD test was used. Values of *n* refer to the number of experiments used to obtain each value. For mouse studies where the data was not normally distributed, we used two-tailed Mann–Whitney *U* test. Values of *p* ≤ 0.05 were considered significant.

## Supplementary information

Suppl Figures 1-5

manuscript checklist

## Data Availability

RNAseq datasets are available from GEO under the accession number GSE204809.
