# Supplementary material for: Thymidine phosphorylase facilitates retinoic acid inducible gene-I induced endothelial dysfunction
Source: Cell Death Dis. 2023 Apr 26;14(4):294. doi: 10.1038/s41419-023-05821-0 (PMC10131517; doi:10.1038/s41419-023-05821-0)
Supplement: Supplementary file 1 — Suppl Figures 1-5 [file 41419_2023_5821_MOESM1_ESM.pdf]

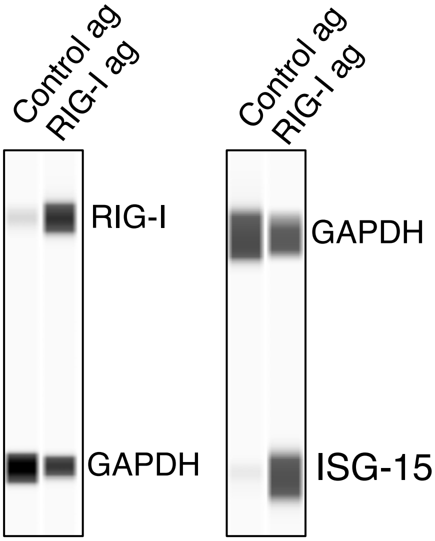

Supplementary Figure 1. RIG-I agonist induces activation of ISG expression. HUVECs were treated with control agonist or RIG-I for 24h. Cell lysates were evaluated for RIG-I and ISG-15 expression using Simple Western Capillary based western blot system (Wes). One of two independent experiments.

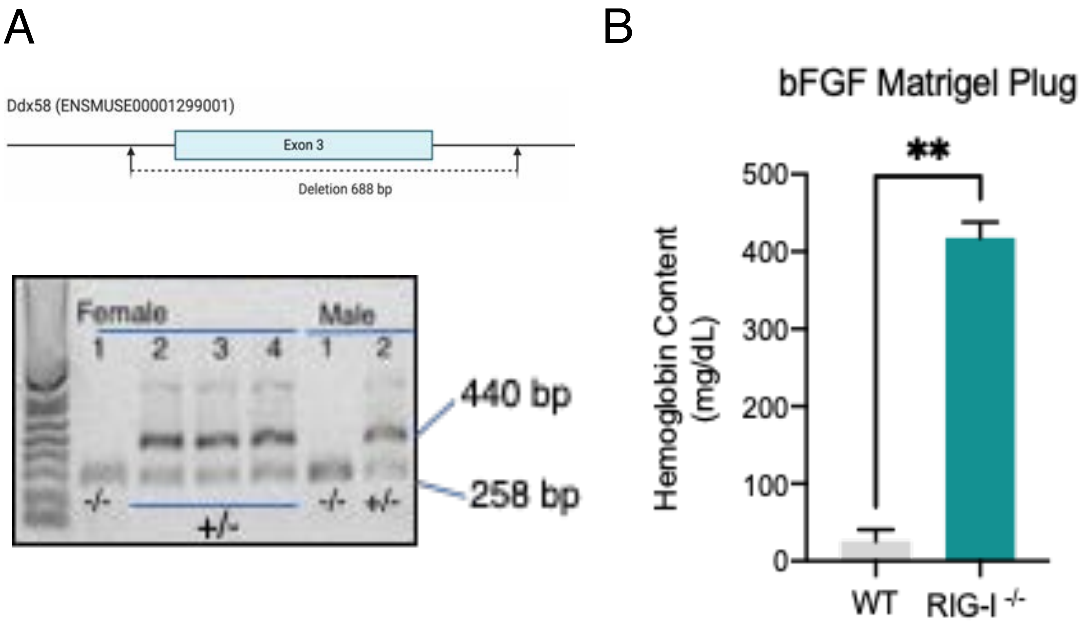

Supplementary Figure 2. Increased hemoglobin content in subcutaneous Matrigel plugs in RIG-I -/- mice. A) Design of 688 bp exon 3 deletion to generate CRISPR edited RIG-I-/- mice and validation of genotypes using tail PCR. B) Hemoglobin content of bFGF containing Matrigel plugs from WT and RIG-I -/- mice on day 7 after subcutaneous implantation (n=4 plugs per group).

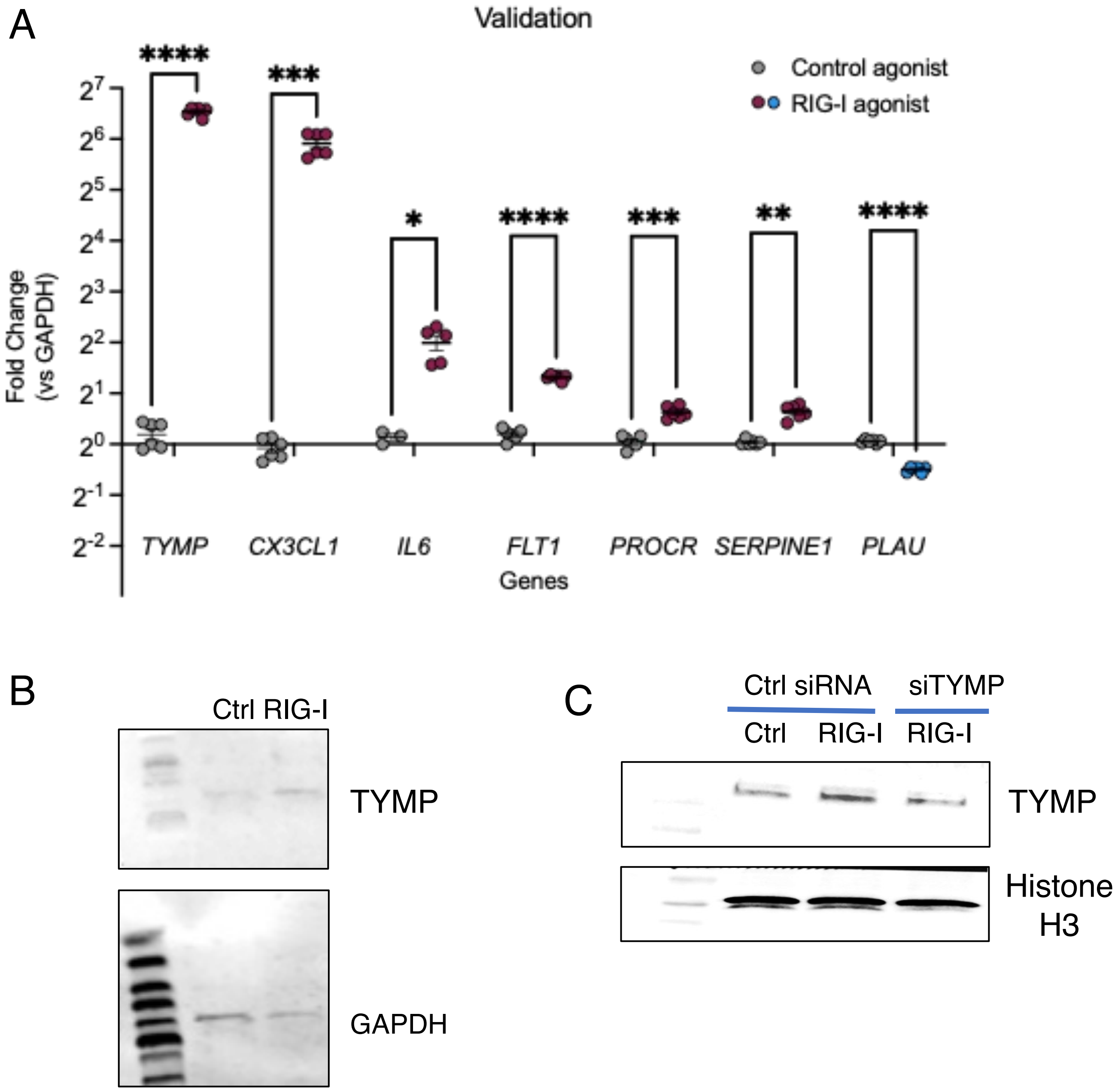

Supplementary Figure 3. Validation of TYMP as a RIG-I induced gene in ECs. A) Gene expression changes of indicated genes 24h after RIG-I agonist or control agonist treatment in HUVECs as measured by qRT-PCR. B) Western blot depicting increase in TYMP protein levels after RIG-I agonist treatment. C) Western blot depicting siTYMP mediated decrease in RIG-I agonist induced TYMP expression.

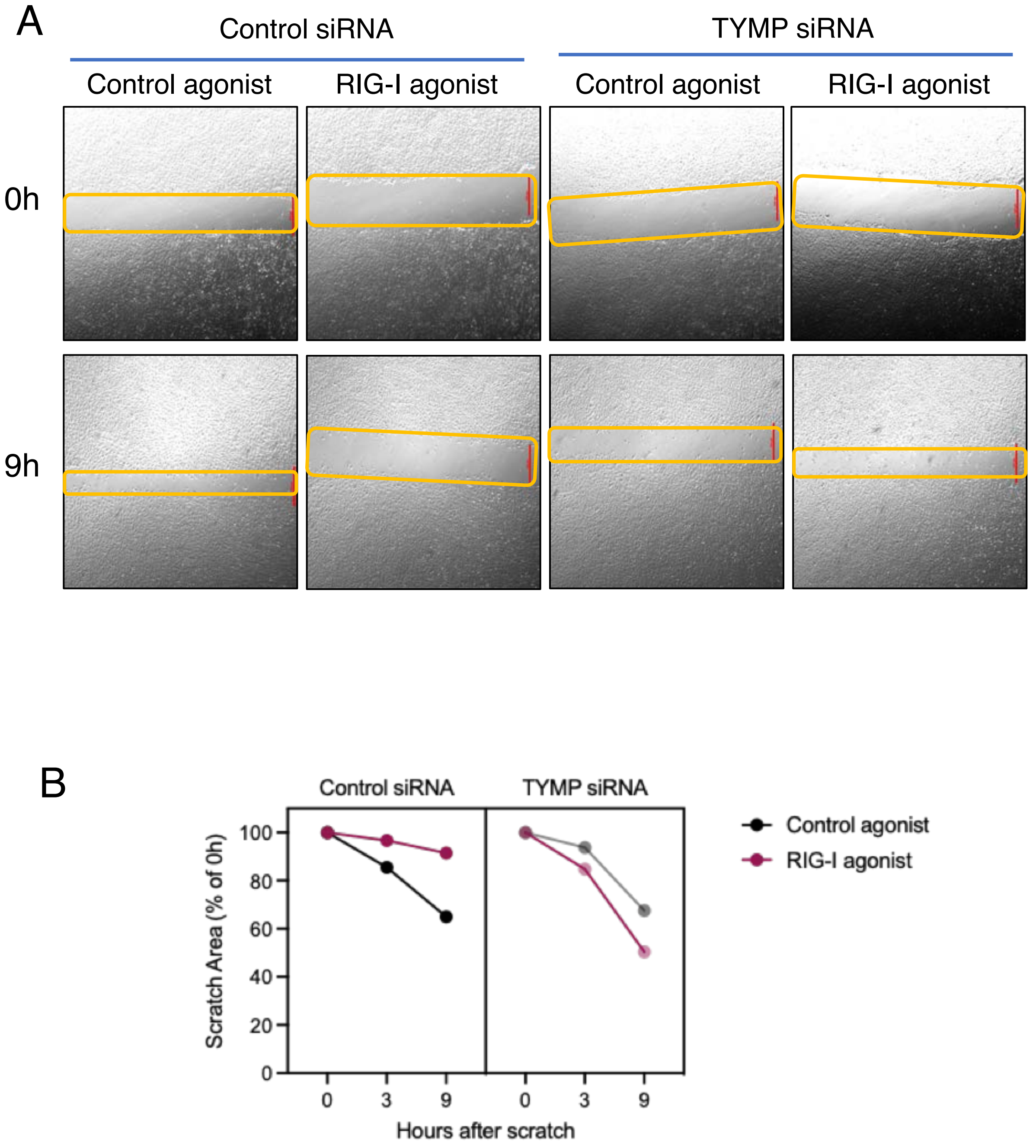

Supplementary Figure 4. Silencing of TYMP with siRNA partially rescues RIG-I induced migration defects in HUVECs. A) HUVECs were transfected with siRNAs as indicated. 16h later, HUVECs were stimulated with either a control agonist or RIG-I agonist as described in Fig 3. Migration was assessed using a scratch assay in a 6-well TC plate. Scale bar = 500 uM. B) Scratch area from images were quantified using Image J. Representative images from one of two independent experiments is shown.

A

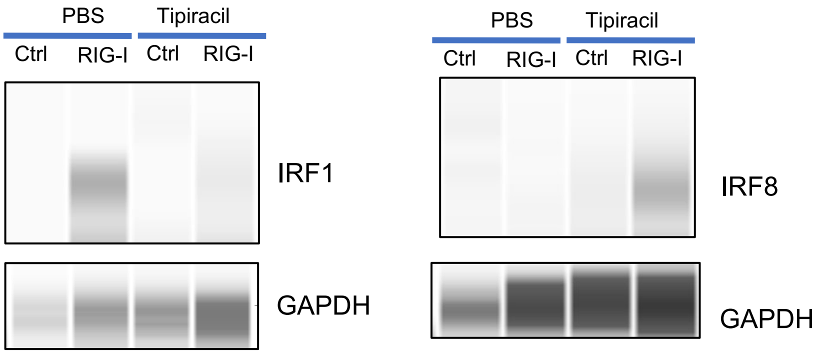

B

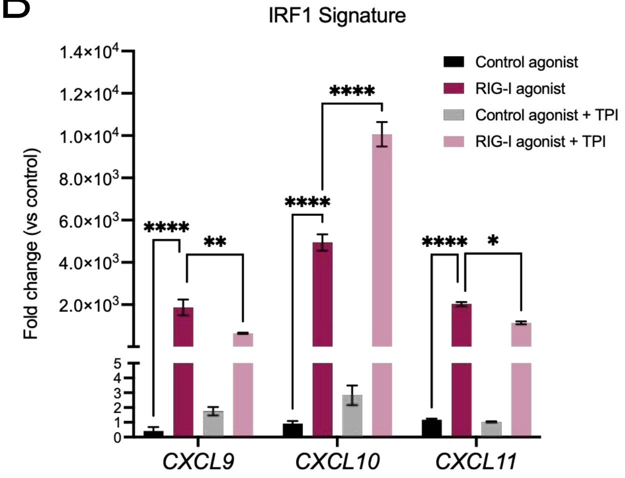

Supplemental Figure 5: Potential regulation of RIG-I induced IRF1 activation by Tipiracil. HUVECs were treated with the Control or RIG-I agonist (0.1 ug/mL) and Tipiracil (10 uM). IRF1, IRF8 levels were assessed by a Simple Western Capillary based western blot (Wes) assay. GAPDH was used as a loading control. B) Transcription of three canonical genes downstream of IRF1 were measured using qRT-PCR. ** P<0.01, *** P<0.005, using ANOVA.
